# Molecular regulation of primordial germ cell specification and implications for in vitro gametogenesis

**DOI:** 10.1093/biolre/ioag188

**Published:** 2026-09-03

**Authors:** Maddison Marshall, Eryl Bevan, Yuan Wang

**Affiliations:** 1 Department of Animal Sciences, College of Agriculture and Natural Resources, Michigan State University, East Lansing, Michigan, USA; 2 Genetics and Genome Sciences Program, College of Natural Science, Michigan State University, East Lansing, Michigan, USA

**Keywords:** primordial germ cells, pluripotent stem cells, mammalian reproduction

## Abstract

Primordial germ cells (PGCs) are the embryonic precursors of gametes, essential for transmitting genetic and epigenetic information across generations. However, PGC specification occurs within a narrow developmental window and involves only a small number of cells, making it difficult to study in vivo. In vitro models using pluripotent stem cells have enabled the generation of primordial germ cell-like cells, but these systems often rely on exogenous signaling and exhibit variability in efficiency and epigenetic fidelity. In this review, we synthesize current understanding of PGC specification in mouse and human systems, emphasizing the integration of signaling pathways, transcriptional networks, epigenetic reprogramming, and metabolic regulation. Canonical regulators, including PRDM1, PRDM14, TFAP2C, and SOX17, function within a broader, interconnected network that establishes for PGC competence. Understanding these interactions will be crucial for advancing in vitro gametogenesis and improving mammalian reproduction.

## Summary Sentence

This paper reviews the interconnected signaling, transcriptional, epigenetic, and metabolic networks that regulate primordial germ cell specification.

## Background

Primordial germ cells (PGCs) are embryonic gametogenic precursors that ultimately give rise to oocytes and sperm, thereby passing genetic information from parent to offspring for species continuity [1]. Disruptions in PGC specification have profound consequences for reproductive health [2]. It is thus critical to elucidate the regulatory network that establishes PGC competence to understand human infertility. Despite their critical role, PGCs are specified during a narrow and early window of embryonic development, when only a small number of cells within the pluripotent epiblast become PGCs [3]. This transient and spatially restricted emergence makes PGC specification inherently difficult to study in vivo. As a result, much of our current understanding remains incomplete, particularly regarding how germ cell fate is established and how cells integrate developmental signals to commit to PGCs.

Recent advances in pluripotent stem cell (PSC)-based models have provided powerful systems to study PGC-like cell (PGCLC) formation in vitro [4–8]. However, these systems often rely on exogenous cytokine-driven induction and exhibit variability in efficiency and fidelity [9], suggesting that key intrinsic regulatory mechanisms remain unresolved. How signaling pathways, transcriptional networks, epigenetic remodeling, and cellular metabolism are coordinated to establish PGC identity is still not fully understood.

In this review, we synthesize current knowledge of PGC specification with an emphasis on the integration of signaling, transcriptional, epigenetic, and metabolic regulation. We highlight emerging evidence that PGC fate is governed by an interconnected regulatory network rather than linear pathways and discuss how resolving these interactions may improve the fidelity and safety of in vitro gametogenesis.

### An overview of human and mouse PGC development

Mouse models have provided a foundational framework for understanding PGC specification, revealing core developmental principles broadly conserved across mammals. Although species-specific differences exist in the timing, signaling requirements, and transcriptional regulators of PGC induction between mouse and human systems, the key steps of PGC induction are similar.In both species, PGCs arise from pluripotent epiblast cells in response to inductive cues from extraembryonic tissues, followed by activation of a largely conserved transcriptional program that suppresses somatic differentiation and initiates epigenetic reprogramming.

Mouse PGCs (mPGCs) are specified around embryonic day 6.25 (E6.25-E7.25) in response to Bone morphogenetic protein (BMP) and WNT signaling from the extraembryonic ectoderm and posterior visceral endoderm. These signals act on pluripotent epiblast cells, which provide a transcriptional state for PGC competence. The canonical transcriptional factors PRDM1 (BLIMP1), PRDM14, and TFAP2C act cooperatively to repress somatic gene programs, promote epigenetic reprogramming, and activate PGC gene networks. This core regulatory network has served as a critical reference for understanding PGC development across species.

Human PGC (hPGC) specification occurs later in development, approximately at day 12 post-fertilization, and relies on a slightly different signaling context and transcriptional network. While BMP signaling remains essential, WNT and Activin/NODAL pathways play stronger roles in establishing competence, and the canonical mouse regulator PRDM14 is largely dispensable in human PSC differentiation models. Instead, SOX17 functions as a principal determinant of hPGC fate, acting upstream of PRDM1 and TFAP2C. SOX17 directly activates PGC-specifying enhancers and represses endodermal differentiation, effectively replacing the role of PRDM14 in the mouse hierarchy. Notably, SOX17 in humans fulfills a role functionally analogous to that of SOX2 in mice. Both factors operate within pluripotent epiblast cells to establish PGC competence and enable activation of a conserved downstream PGC transcriptional network.

Following specification, PGCs sequentially undergo phases of migration, gonadal colonization, proliferation, and sex-specific differentiation that are essential for generating functional gametes. In mice, PGCs migrate from the posterior epiblast through the hindgut to reach the developing gonadal ridges by approximately E10.5.Following an initial G2 arrest, migratory PGCs start to re-enter cell cycle after E9 [10]. PGC number expands from ~150 at E8.5 to > 1000 by E10.5, while maintaining their identity through continued expression of PRDM1, TFAP2C, and pluripotency-associated factors [10, 11]. Upon colonization of the gonad, PGCs undergo rapid proliferation to reach ~25 000 cells at E13.5, complete epigenetic reprogramming, and initiate sex-specific differentiation: in females, germ cells enter meiosis and are arrested at prophase I during fetal development, whereas in males, they undergo mitotic arrest and resume differentiation postnatally. Importantly, PGC development is accompanied by continued epigenetic reprogramming, including imprint erasure and reestablishment, which is essential for generating functional gametes.

While human and mouse PGC specification differ in timing and core regulators, they rely on conserved signaling pathways, transcriptional regulation, and epigenetic programs. Mouse models remain highly informative for understanding the molecular mechanisms governing PGC specification and development, providing critical insights into human germ cell biology and reproduction that would be otherwise inaccessible in vivo.

Because PGCs are rare and transient during embryogenesis in both humans and mice, much of the mechanistic understanding of PGC specification has been derived from in vitro models using PSCs. Across both mouse and human systems, multiple differentiation strategies have been developed to generate PGCLCs, typically by exposing them to combinations of inductive cues such as BMP and WNT signaling. These in vitro systems recapitulate many transcriptional and epigenetic features of endogenous PGCs and have enabled the dissection of the signalling pathways, transcription factors, and chromatin regulators that govern gametogenic fate. Some of the regulatory mechanisms discussed throughout this review, including canonical PGC transcription factors, signaling pathway interactions, and epigenetic reprogramming events, have been discovered or confirmed using these stem cell-based PGCLC models.

### Signaling pathways governing PGC specification

The specification of PGCs is governed by a network of interlinked signaling pathways that integrate extrinsic cues from extraembryonic tissues with intrinsic transcriptional and epigenetic regulators. Among these, BMP, WNT, ERK/MAPK, and NODAL pathways play pivotal roles in establishing PGC competence and ensuring appropriate germ cell development (Figure 1 and 2).

### BMP and WNT Signaling initiate PGC specification

BMP signaling is the canonical and essential pathway initiating PGC specification. In the mouse embryo, BMP4 and BMP8b secreted from the extraembryonic ectoderm, together with BMP2 from the visceral endoderm, act on adjacent proximal epiblast cells between E6.25 and E7.25 to induce PGC precursors. Signal transduction occurs *via* phosphorylation of receptor-regulated SMADs (i.e. SMAD1, SMAD5, and SMAD9), which complex with SMAD4 and translocate to the nucleus to activate key PGC genes including *Prdm1* (*aka Blimp1*), *Prdm14*, and *Tfap2c*. Loss of BMP4, BMP8b, or SMAD1/5 leads to a complete ablation of PGCs, demonstrating the indispensability of this pathway.

WNT/*β*-catenin signaling acts in coordination with BMP to establish PGC competence. WNT3, secreted by the posterior visceral endoderm and neighboring epiblast, is indispensable for forming the BMP-responsive population in which PGCs emerge [12]. Activation of WNT3 induces *Brachyury* (*T*) and *Eomesodermin* (*Eomes*), transcription factors that transiently activate PRDM1 expression in coordination with BMP signals [13, 14]. Precise regulation of WNT signaling is critical: excessive *β*-catenin activation redirects cells toward mesodermal fates, whereas insufficient activity fails to establish PGC competence [13]. BMP and WNT signaling function cooperatively and are both required for PGC specification, as loss of either pathway prevents PGC formation [13].

### Attenuation of ERK/MAPK Signaling stabilizes PGC identity

The ERK/MAPK pathway integrates extracellular signals to control early epiblast lineage decisions. In the pre-gastrulation embryo, ERK activity is high in regions biased toward somatic differentiation, whereas prospective PGCs arise in a localized domain characterized by ERK suppression [12]. In vitro, inhibition of ERK/MAPK signaling enhances PGC induction efficiency from PSCs, consistent with the observation that PRDM14 promotes gametogenic fate by repressing FGF/ERK signaling [5, 15]. PRDM14 actively reinforces the PGC program by attenuating ERK/MAPK signaling to stabilize germ cell identity [15]. These findings indicate that timely attenuation of ERK signaling is essential for stabilizing PGC identity following initial specification.

### Nodal integrates BMP and WNT cues to establish competence for PGC specification

NODAL signaling has also emerged as a regulator of PGC specification. Beyond its established function in primitive streak patterning, epiblast NODAL activity cooperates with BMP and WNT signaling to control PGC competence and the size of the PGC founder population [16]. In mice, genetic disruption of Nodal signaling reduces the number of specified PGCs and alters expression of key PGC regulators [16].

In human PGCLC models, NODAL has emerged as a central determinant of PGC induction downstream of BMP and WNT cues. Rather than acting broadly, WNT signaling primarily functions to induce NODAL, whose precise timing and duration then dictate whether cells adopt PGCLC, amnion-like, or primitive streak-like fates [17].Together, these findings highlight NODAL as a conserved regulator that integrates multiple signaling pathways to direct PGC specification [17].

In summary, these pathways show that PGC specification arises from a highly coordinated network of BMP, WNT, ERK/MAPK, and NODAL signaling. Although the core components required for PGC induction have been defined, the field still lacks a complete understanding of how these pathways are integrated. Dissecting these unresolved interactions remains essential for explaining how germ cell fate is established in vivo and for improving the fidelity and efficiency of in vitro PGCLC generation.

### Canonical and emerging transcriptional regulators of PGC specification

Recent studies have expanded the transcriptional landscape underlying PGC specification, identifying additional genes that act alongside canonical regulators such as PRDM1, PRDM14, and TFAP2C. These newly characterized transcription factors, including NR5A2, ZFP296, TEX10, and RHOX6, modulate chromatin accessibility, WNT signaling, and lineage-specific gene networks to ensure robust PGC induction and subsequent sex-specific differentiation (Figure 1 and 2).

### Canonical transcription factors in PGC specification

Foundational work to understand the transcriptional network underlying mammalian PGC specification has established a core molecular program that drives germ cell fate from pluripotent epiblast cells [18, 19]. Saitou et al. first described the transcription factor STELLA (i.e. DPPA3) as a critical regulator that instructs founder PGCs to escape somatic cell fate and reacquire pluripotency during early mPGC specification [19]. STELLA is thus widely used as a marker of PGC identity during mPGC specification [20]. Subsequent studies *found that Prdm1-null* embryos failed to form PGCs *and* that PRDM1 helps drive the PGC transcriptional program by repressing somatic gene expression while promoting germ cell-associated genes [18, 21]. *Parallel studies then identified PRDM14 as a second transcription factor required for PGC specification and found that Prdm14*-deficient embryos exhibited reduced PGC formation and *impaired epigenetic identity* [22, 23]. TFAP2C emerged as another indispensable transcription factor, in which conditional deletion of *Tfap2c* disrupted early germ cell development and led to the loss of PGCs shortly after specification [24] (Figure 1).

In humans, in vitro models of PGC-like cell (hPGCLC) specification from pluripotent stem cells have enabled mechanistic studies of hPGC fate. In a 2015 study, Irie et al. identified SOX17 as an essential factor of hPGC identity, a role not conserved in mPGCs [7]. While SOX17 remains the central determinant of human gametogenic fate, multiple studies have shown that efficient hPGCLC induction requires prior activation of EOMES, a WNT- and Activin/NODAL-responsive transcription factor that promotes a PGC-competent intermediate rather than a terminal lineage outcome [8, 17, 25]. Functional studies placed PRDM1 downstream of SOX17 in promoting the germ cell fate [7]. Subsequent work confirmed that transcription factors, including TFAP2C and members of the GATA family, such as GATA2 and GATA3, cooperate with SOX17 to establish the hPGC transcriptional program [25]. Notably, in contrast to the mouse, PRDM14 is largely dispensable for hPGC specification, as hPGCLCs can be efficiently induced in its absence, indicating that SOX17-centered regulatory networks function independently of PRDM14 to initiate PGC induction in humans [7, 8] (Figure 1).

These discoveries have enabled the development of transcription factor-driven in vitro PGC differentiation models that bypass exclusive reliance on cytokines. In the mouse, forced expression of PRDM1, PRDM14, and TFAP2C is sufficient to induce PGCLC identity from pluripotent stem cells [14], and these in vitro-derived PGCLCs can undergo complete gametogenesis to generate functional sperm and oocytes capable of producing fertile offspring following transplantation or reconstitution in gonadal environments [5, 6].

### Transcription factors that regulate WNT signaling

Genome-wide CRISPR screening has expanded the transcriptional hierarchy governing PGCLC specification beyond the canonical core regulators PRDM1, PRDM14, and TFAP2C, identifying NR5A2 and ZFP296 as essential, stage-specific regulators of PGC specification [26]. Whereas core transcription factors such as PRDM1/14/TFAP2C act directly to initiate and stabilize PGC identity by repressing somatic programs and inducing germ cell transcriptional networks, NR5A2 and ZFP296 function downstream of, or in parallel with, these factors to enable proper PGC specification. Both NR5A2 and ZFP296 are dispensable for PSC maintenance and epiblast-like cell (EpiLC) formation, yet are strictly required during the transition to PGCLCs, where their loss selectively disrupts PGC specification without broadly impairing somatic differentiation.

NR5A2 and ZFP296 refine PGC competence by coupling transcriptional and epigenetic control to precise signaling outputs. NR5A2 directly regulates *Oct4* through binding to its proximal promoter and enhancer elements, linking the pluripotency network to PGC competence [27–29], while simultaneously restraining excessive WNT/mesodermal signaling during PGCLC induction [26]. In contrast, ZFP296 promotes PGC specification by enabling appropriate WNT-dependent activation of PRDM1, PRDM14, and TFAP2C, and by antagonizing H3K9 methylation to support the extensive chromatin reprogramming required for PGC development [30, 31]. Together, these studies show that NR5A2 and ZFP296 are critical regulatory factors that fine-tune WNT signaling and chromatin state to ensure faithful deployment of the core PGC transcriptional program.

Following acquisition of PGC competence, transcription factors integrate extrinsic signaling cues to activate the PGC transcriptional program. One of the earliest regulators in this process is the mesodermal transcription factor T/BRACHYURY. T/BRACHYURY is transiently expressed in the posterior epiblast and primitive streak, where it functions downstream of WNT signaling. PGCs arise from cells that transiently express T/BRACHYURY prior to specification [13]. T/BRACHYURY directly activates key PGC determinants, including PRDM1 and PRDM14, thereby initiating the transcriptional cascade that drives PGC specification. Loss of T/BRACHYURY impairs PGC formation, whereas its ectopic activation promotes germ cell-specific gene expression under permissive signaling conditions, identifying T/BRACHYURY as a key link between WNT signaling and PGC transcriptional activation [13].

The testis-expressed gene 10 (TEX10) has recently emerged as another important regulator of PGC development, acting at the intersection of pluripotency maintenance and WNT pathway control. TEX10 encodes a transcription factor that has been implicated in PSC maintenance and transcriptional elongation. TEX family gene variants have been linked to human azoospermia and male infertility, though knockdown of *Tex10* in Drosophila resulted in normal fertility [32]. Functional studies demonstrate that TEX10 is required for efficient PGCLC induction in vitro and for proper spermatogenesis in vivo, with loss of *Tex10* leading to reduced PGCLC formation, increased apoptosis, and defective male germ cell maturation [32]. TEX10 directly binds to and activates WNT-negative regulators such as PSMD3 and PSMD7, restraining excessive WNT/*β*-catenin activity that would otherwise divert cells toward somatic lineages. TEX10 depletion results in hyperactivated WNT signaling, impaired PGC specification, and dysregulation of WNT targets across both PGCLCs and developing spermatids. Conversely, TEX10 overexpression enforces WNT repression, maintains pluripotency networks, suppresses somatic gene programs, and markedly enhances PGCLC induction [32]. Together, these findings demonstrate TEX10 is a critical modulator of gametogenic fate, coordinating transcription factor activity with precise restraint of WNT signaling to ensure proper PGC specification and male fertility.

### Transcription factors critical for PGC dormant totipotency

During early embryonic development, pluripotent cells transition from a naïve to a primed state, accompanied by down-regulation of core pluripotency factors [5]. To acquire PGC competence, a subset of these cells must re-establish key features of pluripotency, while activating the transcriptional program required for PGC specification and repressing somatic differentiation programs.

Among these factors, NANOG plays a central role in establishing PGC competence by activating key PGC determinants while suppressing epiblast differentiation cues [33].In the preimplantation embryo, NANOG sustains naïve pluripotency, but its expression diminishes as cells transition to the EpiLC state [34]. Remarkably, transient re-expression of NANOG is sufficient to induce PGCLCs even in the absence of cytokines, demonstrating that NANOG can bypass canonical extrinsic signaling requirements [33]. NANOG binds and activates enhancers of *Prdm1* and *Prdm14*, two master regulators that initiate the PGC regulatory network [33].

By contrast, OTX2 acts as a transcriptional repressor that defines the boundary between somatic and PGC developmental trajectories [35]. OTX2 is highly expressed in the primed epiblast and directly represses essential PGC determinants, including *Nanog* and *Prdm1* [35, 36]. Downregulation of OTX2 is therefore a prerequisite for acquiring PGC competence. Conversely, NANOG antagonizes OTX2 in promoting somatic lineage specification. Depletion of OTX2 leads to premature and ectopic PGC formation in mice, whereas its sustained expression suppresses PGC induction even in the presence of BMP signaling [35].

The transcription factor ESRRB cooperates with NANOG to reinforce a naïve pluripotency transcriptional state and promote responsiveness to PGC-inducing signals [37].ESRRB is a direct target of LIF/STAT3 signaling and critical for maintaining pluripotency in naïve ESCs [38]. Induced expression of ESRRB in the absence of NANOG restores PGC numbers in vitro and in vivo [39]. Although, ESRRB alone cannot induce PGC transcription factors BLIMP1 and PRDM11 in the absence of cytokines, but successfully induced these factors in OTX-null ESCs [38, 40]. Overall, these findings highlight that PGC specification is controlled by a balance among the PGC activators NANOG and ESRRB and the PGC suppressor OTX2. Precise regulation of this balance is critical for the proper timing of PGC specification and segregation from somatic fate.

Additional regulators further coordinate the maintenance of pluripotency with PGC induction. The reproductive homeobox transcription factor RHOX6, a member of the X-linked *Rhox* gene cluster, has recently emerged as a regulator of both PSC self-renewal and PGC specification. RHOX6 is expressed in undifferentiated PSCs and becomes dynamically upregulated during PGCLC specification [41, 42]. Functional studies demonstrate that RHOX6 promotes PGC specification by enhancing PGCLC formation and upregulating key PGC regulators, including *Prdm14, Tfap2c, Stella,* and *Nanos3* [42]. RHOX6 directly binds to the *Nanos3* promoter, and depletion of *Nanos3* attenuates the ability of RHOX6 to promote PGCLC formation. Interestingly, RHOX6 also regulates pluripotency dynamics, as loss of *Rhox6* enhances PSC proliferation and naïve pluripotency, partly through repression of the transcription factor TBX3 [42]. These findings suggest that RHOX6 coordinates the transition from pluripotency toward germ cell differentiation by simultaneously promoting PGC gene transcription and limiting excessive self-renewal.

### Transcription regulators critical for the proliferation and survival of PGCs

Before sex-specific differentiation, PGCs must migrate to embryonic gonads and continue to proliferate while maintaining their identity. These processes are governed by additional regulators that stabilize the germ cell transcription program, maintain PGC survival, and protect them from somatic differentiation. Among them, OCT4 (POU5F1) is a key pluripotency factor highly expressed in both PSCs and PGCs. In mice, OCT4 is abundantly expressed in the inner cell mass of the blastocyst but becomes progressively downregulated in the epiblast, remaining restricted to newly specified PGCs by E7.5 [43, 44]. Genetic ablation of *Oct4* by a PGC-specific *Tnap-Cre* driver results in apoptosis of existing PGCs rather than differentiation into trophectoderm, highlighting its essential role in maintaining PGC survival [45]. In humans, OCT4 has been shown to switch its transcriptional partners from SOX2 to PAX5 and PRDM1 during PGCLC differentiation from PSCs, thereby facilitating the appropriate progression of the germ cell program by repressing somatic gene expression [46].

RNA-binding proteins from the DAZ (Deleted in Azoospermia) family also play critical roles in regulating germ cell survival, proliferation, and long-term developmental competence. The DAZ family contains three members: BOULE, DAZL (Deleted in Azoospermia-Like),and DAZ [47].BOULE is the most ancient member and is expressed across many species, including worms, rodents, and humans, whereas DAZ is found only in primates. BOULE and DAZ appear to function in meiotic and post-meiotic stages of germ cell development. By contrast, DAZL is a highly conserved germ cell-specific protein in vertebrates and becomes robustly expressed as PGCs colonize the gonadal ridges [48–52]. DAZL was initially identified through its association with human infertility, and further functional studies in mice established DAZL as a central regulator of germ cell development by limiting somatic differentiation [47, 50]. Although PGCs are specified normally, loss of *Dazl* reduces PGC numbers due to increased apoptosis and impaired progression through germ cell developmental programs [48, 52, 53]. DAZL acts post-transcriptionally to promote the translation of key germ cell-specific mRNAs, including those involved in meiotic entry and differentiation [54–56]. DAZL may also promote hPGC development by regulating microRNA maturation [57]. Ectopic expression of DAZL enhances the formation of PGCs from both human and mouse PSCs [49, 57, 58]. Several other germ cell-specific genes, including GASZ, coordinate with DAZL to promote PGC development and will be discussed below.

### Epigenetic regulation during PGC specification

PGC specification is not solely a cell-signaling and transcription-factor-driven event but also an epigenetic reprogramming that defines cellular identity. In mammals, germ cell is established within the epiblast, which is transcriptionally plastic but epigenetically constrained. To commit to the germ cell fate, nascent PGCs must remove somatic epigenetic programs while simultaneously installing a germ cell epigenetic program (Figure 2).Thus, epigenetic reprogramming is not a secondary consequence of PGC specification; it is a core regulatory mechanism that enables and stabilizes germ cell identity.

### Coordinated DNA methylation machinery enables PGC specification

Mammalian genomes primarily exhibit methylation at CpG dinucleotides as 5-methylcytosine (5mC) [59]. The global methylation level is a net balance between methylation catalyzed by DNA methyltransferases (DNMTs) and demethylation by Ten-Eleven Translocation (TET) family enzymes [59]. mPGC precursors at E6.75 exhibit methylation identical to their somatic neighbors in the epiblast [10, 60], but as PGCs migrate through the hindgut and dorsal mesentery to the embryonic genital ridge, they undergo genome-wide demethylation, colonizing the genital ridge around E10.5–11.5 and reaching ~4% methylation in E13.5 PGCs [61, 62]. This global erasure is achieved through both passive and active demethylation [63], essential for eliminating somatic epigenetic memory, resetting genomic imprints, and remodeling regulatory elements required for germ cell-specific gene expression.

### Passive demethylation of the PGC genome

PGC-specific genome-wide demethylation occurs in two phases. The first phase (E7.5–10.5) is dominated by passive demethylation, during which methylation marks are not maintained in DNA replication and thus progressively diluted with cell division [64, 65]. During this period, *de novo* DNA methyltransferases DNMT3a/3b are downregulated [10, 60]. Critically, repression of DNMTs is regulated by core PGC transcription factor PRDM14, highlighting an intertwined transcriptional and epigenetic regulatory mechanism [66]. Maintenance methyltransferase DNMT1 acts on hemi-methylated DNA [67, 68] but depends on UHRF1 for recruitment to S-phase replication forks [69–71]. UHRF1 expression decreases significantly during PGC formation, with the remaining protein being excluded from the nucleus [60, 72], thereby preventing DNMT1 from assessing DNA for methylation. Imprinting control regions (ICRs), germ cell-related genes, and CpG islands on the X chromosome are less affected by demethylation during this phase [73]. Meiotic gene promoters also retain methylation before E10.5 to prevent premature meiosis in females and differentiation in males [73]. These regions are primarily demethylated by TET-mediated active demethylation.

### TET enzymes actively demethylate the PGC genome

Active demethylation in the second phase is mediated by methylcytosine dioxygenases TET1, TET2, and TET3. TET family enzymes share a conserved catalytic core that oxidizes 5mC to 5hmC. In addition to permitting active removal of methylation, TET enzyme action also facilitates passive demethylation as 5hmC is not efficiently recognized by DNMT1, which normally recognizes 5mC to preserve methylation patterns [74].

Beginning at E10.5, as PGCs colonize the genital ridge, TET1 and TET2 mediate genome-wide DNA demethylation, including parentally imprinted loci and demethylation-resistant promoters, resulting in approximately 96% of the genome becoming hypomethylated [75, 76]. TET1 and TET2 are expressed during PGC migration and subsequent colonization of the gonad [77, 78]. TET1 is expressed at a higher level than TET2 at this time. Parentally imprinted genes are often organized in clusters associated with ICRs, where DNA methylation on one parental allele is maintained and resistant to passive demethylation. In PGCs, imprinted loci exhibit an increase in 5hmC marks concomitant with the decrease in 5mC marks, indicating that TET-mediated oxidation is a likely mechanism to demethylate these ICRs [75, 79]. In addition, similar hypermethylation in ICRs was observed in *Tet1* null and *Tet1/2* compound knockout male mice, indicating that TET1 is the chief contributor to the demethylation of ICRs during PGC migration [74, 75, 80].

By E13.5, the PGC genome is almost entirely demethylated, including ICRs, but not some retrotransposons and female germ cell-specific genes [73].After this point, male and female PGCs develop sex-specific methylation in response to gonadal cues: female PGCs remain globally hypomethylated (4–7%) as they enter meiosis, whereas male PGCs enter mitotic arrest, with methylation increasing to 50–60% by E16.5 [60, 62].

### Histone modifications in PGC development

In addition to demethylation during PGC development, concurrent histone modification processes occur. Mouse PGCs exhibit a distinct chromatin landscape characterized by histone H3 lysine 9 (H3K9) hypomethylation and H3K27 hypermethylation [10, 60, 62]. In mammalian cells, 50% of histone H3K27 trimethylation (H3K27me3) peaks overlap with promoters and gene bodies, while 75% of H3K9me3 peaks occur in intergenic regions [81]. Repressive H3K9 dimethylation (H3K9me2) is globally reduced while permissive/activating histone modifications H3K4me2, H3K4me3, and H3K9ac, and repressive H3K27me3 are increased in E8.5 PGCs [10]. This increase in H3K27me3 marks may compensate for the loss of H3K9me2 marks and DNA methylation during PGC migration [10, 82].

Histone modifications continue to be dynamically changing during PGC migration (E8.5–E11.5) and gonadal colonization. H3K9me3 and histone H1 levels are initially reduced during migration and subsequently re-established following PGC colonization to the genital ridges [82]. In contrast, H3K27me3 is transiently enriched during migration but declines during later gonadal stages (E10.5–E13.5), particularly in male PGCs [81]. Active chromatin marks such as H3K9ac and the histone variant H2A.Z are also markedly reduced during reprogramming and remain low during this developmental window. However, Kagiwada et al. disputes these H1 and H3K27me3 observations [83]. Additional histone modifications associated with transposable elements (TEs) also undergo dynamic changes. For example, H3K64me3 modifications, associated with IAPs and LINE1 transposable elements, are reduced at E12 and re-established by E13.5 [84].

Following colonization of the genital ridge, histone modifications exhibit pronounced sex-specific dynamics. Between E10.5 and E13.5, global H3K27me3 levels decline, with a more substantial reduction in male PGCs [81]. H3K27me3 is deposited by PRC2, which comprises the core components EED,SUZ12,and the catalytic methyltransferase EZH2.Conditional knockout of *Eed* resulted in severe loss of H3K27me3 and reduced PGC numbers, beginning at E11.5 in XX and E12.5 in XY embryos [85]. Nevertheless, PRC2 activity may not be strictly required for the initial PGC specification, as chemical inhibition of EZH1/2 or deletion of *Ezh2* showed no reduction of PGCs before E13.5 [81, 86, 87]. After E13.5, *Ezh2* knockout leads to loss of H3K27me3 without affecting H2AK119ub (monoubiquitinated H2A at lysine 119) or H3K9me3 and results in more pronounced functional defects in female PGCs [81], reflecting their greater reliance on PRC2-mediated repression to compensate for a persistently hypomethylated state. During this same period, H3K9me3 is differentially regulated: levels remain relatively stable in female PGCs but increase substantially in male PGCs. These changes are not due to sex specific-expression of histone methyltransferases, but rather differential expression of PRC2 auxiliary components AEBP2 and PHF19, which may influence chromatin organization during epigenetic reprogramming [81].

Notably, PGCs also share key chromatin features with pluripotent stem cells, including H3K27ac marks at genes that maintain an undifferentiated state [88]. Repressive H3K27me3 marks are enriched at lineage-specific developmental regulators, preventing somatic differentiation [89]. In addition, many lineage-specific genes in PGCs exhibit bivalent histone marks, i.e. they are simultaneously marked with active H3K4me3 and repressive H3K27me3. These genes, including *hox* genes, are repressed yet hypomethylated, indicating they are poised to be activated. During PGC specification, many of these bivalently marked genes lose H3K4me3, reinforcing transcriptional repression of somatic programs [90].

Human PGCs share several key features of chromatin reprogramming with mPGCs, including global loss of H3K9me2 and dynamic regulation of H3K27me3, although the extent and timing of these changes differ between species. In hPGCs, H3K27me3 increased during early stages and then substantially reduced during the imprint erasure stage [91, 92]. Compared to gonadal somatic cells, hPGCs were also enriched for neutral and permissive chromatin marks H3K4me1, H3K4me3, and H3K9ac, peaking at embryonic week 10–13 [91, 92]. In addition, in hPGCs, repressive chromatin is organized into two broad groups: those occupied by H3K9me3 and 5mC and those marked by H3K27me3 and H2aK119ub [92]. However, hPGCs exhibit lower H3K27me3 and H2aK119ub relative to mPGCs [92]. Sex-specific chromatin differences are also observed in hPGCs. Male hPGCs show increased H3K4me3 at later stages, whereas female hPGCs exhibit distinct chromatin features associated with X chromosome reactivation, with enriched H3K4me3 signals at CpG island promoters [93].

### Repression of transposable elements safeguards PGC genome

Transposable elements are genomic sequences derived from ancient viruses or mobile DNAs that invade the genome during evolution. In PGCs, TE repression is maintained through DNA methylation, repressive histone marks (e.g. H3K9me3 and H3K27me3), and piRNA-mediated pathways, thereby minimizing TE expression during epigenetic reprogramming.

During PGC development, H3K9me3 and DNA methylation exhibit a strong positive correlation at CpG-rich regions [94, 95]. Compared with nearby unmethylated regions, both DNA methylation and H3K9me3 are concentrated at the co-marked CpG-rich regions (CHMs), a pattern unique to PGCs but not in other lineage development [95]. CHMs are enriched in TEs, particularly evolutionarily young LTR families such as ERVK [95].When DNA methylation is low at E13.5, a subset of ERV and LINE1 transposable elements are marked with H3K9me3 and H3K27me3. H3K27me3 marks are enriched at older and inactive TEs, while H3K9me3 marks are enriched at younger TEs with potential for transactivation, suggesting a key role for H3K9me3 in TE silencing [96]. H3K9 methyltransferase SETDB1 plays a critical role in these histone modifications and deposits H3K9me3 marks at TEs in a KAP1-dependent manner [92]. For a subset of ERV and LINE1 TE families, H3K9me3 marks are dependent on SETDB1, disruption of which is sufficient for de-repression, despite these regions being co-enriched for H3K27me3.

Similar regulatory mechanisms are observed in humans. 5mC and H3K9me3 marks were the most prominent at TEs in hPGCs, although only ~38.6% of TEs retain repressive marks in female PGCs, compared to 97.2% in gonadal somatic cells [92]. A global loss of repression is also observed in male hPGCs. Many TEs are bivalently marked, defined as H3K4me3 together with a repressive modification.H3K9me3 bivalently marked TEs include young and active ERVK, SVA, and ERV1 TEs, while 5mC bivalently marked TEs (lacking H3K9me3) include Alu and SVA TEs. H3K27me3 and H3K9me3 co-marked TEs are the most strongly repressed [92]. While DNA methylation escapee regions are primarily marked by H3K9me3, H3K27me3 is also present. These repressive histone modifications are crucial for restraining TEs from activation through global DNA demethylation and local H3K4me3 signals [92, 93].

The mature piRNAs (Piwi-interacting RNAs) are a class of 24–34 nucleotide-long, small non-coding RNAs that associate with PIWI (P-element-induced wimpy testis) family proteins. PiRNAs are predominantly expressed in germ cells and provide an additional layer of regulation for TE repression in both humans and rodents [97–102]. Mature piRNAs are generated from long single-stranded precursor transcripts that are processed through a mitochondria-associated biogenesis pathway [97–102]. In embryonic germ cells, more than half of piRNAs originate from TEs or their remnants [97–102]. These piRNAs guide PIWI proteins to complementary TE transcripts, leading to their post-transcriptional cleavage in the cytoplasm. PIWI–piRNA complexes also recruit chromatin modifiers in the nucleus to deposit repressive marks, such as H3K9me3 and DNA methylation, at TE loci, thereby reinforcing their transcriptional silencing [103, 104]. The piRNA pathway is activated at E12.5 and onward during late gonadal development [97–102]. This is particularly important as global DNA demethylation has erased many epigenetic barriers to TE activation. Although repressive histone modifications, such as H3K9me3, provide partial protection against TE expression during this hypomethylated state, the piRNA pathway acts as a complementary and essential genome surveillance mechanism to preserve genome integrity. Interestingly, although the piRNA pathway is required for both male and female reproduction in most species, including humans, it is dispensable in mouse ovarian development (reviewed in [105]). One proposed explanation is the expression of the oocyte-specific DICER isoform/DICER(O) in mice, which generates endogenous siRNAs to suppress TE activation [106–108]. However, combined disruption of *Dicer*^*O*^ and piRNA pathway regulator (i.e. *Mili*) did not impair mouse ovarian development [109], suggesting that additional mechanisms contribute to transposon silencing in the female germ cells.

### Germ cell-specific regulators coordinate epigenetic modifiers during PGC development

Core PGC regulators may directly interact with epigenetic regulators to reset the epigenetic program for germ cell development. For example, PRDM1 selectively recruits HDAC3 to somatic genes, deacetylating nearby histones to repress somatic differentiation [110]. BMP4 also represses somatic differentiation but shares only a few targets with HDAC3 and largely represses independent gene sets. Most of those shared targets are associated with early embryogenesis (e.g. *hoxa1/b1/a2/b2, pax6,* and *lhx1*), whereas genes for specific somatic lineages are repressed independently [111]. *Hdac3* knockdown significantly downregulates PGC determinant genes, including *T, Prdm1, Prdm14, and Tfap2c*, as well as two early PGC markers (i.e. *Stella and Nanos3*) and *Rhox6/Rhox9* during later PGC development. By contrast, somatic markers including *Hoxa1* and *Hoxb1* are elevated upon *Hdac3* knockdown. *Hdac3* knockdown also results in a significant loss of PGCs, a phenotype similar to *Prdm1* knockout. Loss of *Hdac3* does not appear to affect BMP/SMAD signaling as p-SMAD1/5/8 proteins are appropriately localized in proximal epiblast [111]. In addition, PRDM1 associates with PRMT5, an arginine-specific histone methyltransferase, to induce dimethylation of arginine 3 on H2A and H4 tails, helping repress the activation of somatic genes [112].

STELLA also plays a role in regulating DNA methylation in PGCs. It is a maternal factor that is zygotically expressed and reappears during PGC specification [113]. In zygotes, STELLA is a negative regulator of TET enzyme function and protects both certain maternally and paternally imprinted loci from premature demethylation [113, 114]. STELLA contains a conserved N-terminal DNA-binding domain [115], which recognizes H3K9me2 histone marks [114] and directly interacts with both TET2 and TET3, suppressing their enzymatic activity in vitro and protecting surrounding DNA methylation [116]. The C-terminus of STELLA protein is required for the exclusion of TET3 from the maternal pronucleus [114]. However, STELLA regulates DNA demethylation independent of TET1 and DNMT enzymes [65, 116]. PGC specification, migration, and proliferation are largely intact in *Stella* knockout mice. The overall methylation patterns were also similar between the wildtype and the *Stella* mutants [65]. This is not entirely surprising, given that TET1 is the major player in active demethylation in PGCs, while STELLA blocks demethylation largely through interactions with TET2 and TET3,both of which have relatively low expression levels [63, 78]. Nevertheless, *Stella* knockout PGCs between E11.5 and E16.5 exhibited methylation levels 1.5–1.8 times higher than those of heterozygous controls. Certain regions that are normally protected (such as imprinted loci, transposons, or repetitive elements) are prematurely or excessively demethylated, suggesting that STELLA also blocks DNA demethylation in PGCs *via* TET-independent mechanisms [117, 118].

Additional studies show that when the PGC genome is demethylated, the level of NRF1,a methylation-sensitive transcription factor, begins to increase [119], potentially leading to further activation of TEs at demethylated loci [120]. Nevertheless, epigenetic screenings and analyses reveal that the promoters of several genes involved in piRNA biogenesis, including *Gasz* (*aka*. *Asz1*), *Mov10l1,* and a PIWI family member, *Mili* (*Piwi2*), are predominantly regulated by DNA methylation rather than repressive histone modifications such as H3K27me3, H3K9me2, H3K9me3, or H4K20me3 [121, 122]. Genome-wide demethylation in post-migratory PGCs thus releases these genes from suppression [121, 122]. NRF1 also binds to the demethylated promoter of *Gasz* to further induce its expression [121]. GASZ is a germ cell-specific protein that self-interacts at the outer mitochondrial membrane [123–125]. GASZ self-interaction subsequently clusters mitochondria into unique subcellular granules, known as intermitochondrial cement (IMC) [126], where GASZ proteins directly recruit MILI for piRNA biogenesis, safeguarding the integrity of the PGC genome against transposon disturbance during late germ cell development [127].In addition to its role in the piRNA pathway, GASZ cooperates with DAZL to promote PGCLC derivation from PSCs [128], suggesting a function beyond transposon repression. It is worth noting that the function of NRF1 is multifaceted. NRF1 also regulates mitochondrial respiration, and its high expression favors mitochondrial metabolism [129], a preferred bioenergetic pathway in PGCs. Conditional deletion of *Nrf1* in germ cells thus compromises PGC proliferation and survival [119]. Together, these findings illustrate how DNA demethylation not only increases the risk of transposon activation but also coordinates the induction of metabolic regulators and the piRNA machinery, linking genome-wide epigenetic reprogramming to metabolic, transcriptional, and post-transcriptional mechanisms that preserve germline genome integrity during PGC development.

### Epigenetic and metabolic processes regulate one another in PGC specification

Lineage specification is accompanied by dynamic metabolic remodeling as developmental cues can alter metabolic enzyme expression and activities, while the extracellular environment may limit metabolite availability. The altered levels of metabolites from different pathways, in turn, impact cell fate decisions *via* regulating epigenetic status and post-translational protein modification. As genes in metabolic pathways are not exempt from epigenetic control, epigenetics and metabolism regulate one another in a two-way street throughout germ cell differentiation.

Several metabolites can directly regulate the activities of epigenetic modulators critical for germ cell development. For example, *α*-ketoglutarate (*α*-KG), a tricarboxylic acid (TCA) cycle intermediate, functions as a cofactor for TET family enzymes and Jumonji C (JmjC)-domain histone demethylases, promoting DNA and histone demethylation [130]. By contrast, related metabolites such as succinate and fumarate can inhibit these enzymes, thereby opposing demethylation [131]. Increases in *α*-KG correlate with lower expression of DNMT3A/3B, as well as an increase in H3K27me3 and a decrease in H3K9me3 [132]. Acetyl-CoA serves as the donor for histone acetylation, linking glycolytic, lipid, and mitochondrial metabolism to chromatin accessibility, while S-adenosylmethionine (SAM), generated through one-carbon metabolism, provides methyl groups for DNA and histone methylation [133].

Early work from 1977 first demonstrated a predominance of aerobic respiration and relatively low glycolytic activity in E15 mouse fetal germ cells compared with fertilized eggs [134]. More recent quantitative metabolomic and proteomic analyses by Hayashi et al. found increased mitochondrial metabolism in E13.5 PGCs, a distinct feature from PSCs or gonadal somatic cells [135]. Metabolic perturbations by rotenone, an electron transport chain inhibitor, affected PGC survival and reduced PGCLC induction from PSCs [90, 135]. Similarly, untargeted metabolomics revealed elevated metabolites from the TCA cycle in PGCLCs [136]. Interestingly, *α*-KG from the TCA cycle appeared to stimulate PGCLC fate specification, at least in part, by increasing the activities of TET1 and H3K9me2 demethylases, KDM3A and KDM3B [137]. Another study showed that *α*-KG addition promoted PGCLCs *via* elevating H3K27me3 levels during PSC differentiation [136]. Similar metabolic regulation is also noted in hPGCLC specification. Supplementation with vitamin C, which enhances the activities of JmjC histone demethylases and TET proteins, increased the levels of 5hmC, leading to activation of key genes involved in hPGC development, including NANOS3, TFAP2C, BLIMP1, and SOX17 [138]. Together, these findings highlight how metabolic flux can modulate the epigenetic landscape to impact germ cell fate specification (Figure 2).

### Generating primordial germ cell-like cells in vitro

Early efforts to model germ cell development in vitro were enabled by foundational advances in pluripotent stem cell culture, including the derivation of mouse embryonic stem cells (ESCs), human ESCs, and induced pluripotent stem cells (iPSCs) [139–144]. These systems provided a platform to investigate developmental processes, including PGC specification, under controlled conditions.

Initial studies in the mouse system provided proof of principle that germ cell fate could be induced from pluripotent cells in vitro using three-dimensional embryoid body-based differentiation systems, which permit spontaneous cell–cell interactions and partial recapitulation of embryonic signaling environments. Toyooka et al. first demonstrated that mouse ESCs cultured in embryoid bodies could be induced toward PGC-like identities through BMP-dependent differentiation, revealing that key PGC inductive signals can be recapitulated outside the embryo [4]. Geijsen et al. extended this work by showing that ESC-derived PGCLCs could undergo further germ cell differentiation, including meiotic progression, highlighting the developmental potential of in vitro-generated germ cells [145]. These PGCLCs exhibit features associated with post-migration PGC in vivo, including partial imprint erasure and expression of late germ cell markers such as DAZL [128, 146].

While embryoid body based systems established an important conceptual framework, limitations in efficiency, heterogeneity, and reproducibility led to the development of more defined and stepwise differentiation strategies.A key advancement was the recognition that PGC competence is a transient developmental state. This led to protocols in which naïve ESCs are first transitioned into EpiLCs before exposure to PGC inducing signals [5]. This approach enables robust and reproducible generation of PGCLCs that closely resemble early embryonic PGCs at transcriptional and epigenetic levels, and that are capable of contributing to functional gametogenesis in vivo [5, 6].

Subsequently, Nakaki et al. introduced a complementary transcription factor-driven approach that further refined the in vitro generation of PGCLCs [14]. In this system, ESC-derived EpiLCs are induced to express a defined set of PGC regulators, PRDM1, PRDM14, and TFAP2C, enabling rapid and efficient conversion into PGCLCs in the absence of exogenous cytokines. Notably, PRDM14 alone was sufficient to initiate a PGC-like transcriptional program in EpiLCs, although the combined expression of all three factors yielded higher-fidelity PGCLCs with more complete epigenetic reprogramming [14]. These transcription factor-induced PGCLCs recapitulate key transcriptomic and epigenetic features of endogenous nascent PGCs and are competent for germline contribution in vivo, including spermatogenesis and the production of fertile offspring [14].

Efforts to model human germ cell development in vitro build on earlier studies of PGCs in human embryos, which established their molecular identity and developmental timing [147], together with advances in human PSC derivation and culture [141, 143, 144]. From these foundational studies, multiple groups reported that PSCs can be directed toward hPGCLCs under defined signaling conditions, thereby providing systems to study early human germ cell development [7, 8, 148]. Irie et al. demonstrated that primed human PSCs could be converted back to the naïve state and then induced to differentiate toward PGCLCs by BMP4. They identified SOX17 as a critical upstream determinant of hPGC fate, revealing a transcriptional hierarchy distinct from that of mice [7]. Sasaki et al. showed that efficient hPGCLC induction requires passage of primed human PSCs through an incipient mesoderm-like, PGC competent intermediate established by WNT and Activin/NODAL signaling prior to BMP exposure, highlighting the importance of developmental competence in hPGC specification [8]. Sugawa et al. further refined these approaches using human induced pluripotent stem cells and optimized cytokine combinations to improve induction efficiency and reproducibility [148]. These approaches are particularly valuable for dissecting regulatory mechanisms and developmental paradigms underlying hPGC specification, which are otherwise inaccessible for study. Using hPGCLC models, DMRT1 was found to play a critical role in epigenetic resetting and transcriptional regulation of germ cells [149]. Similarly, KLF4 was identified to promote hPGCLC development by activating genes involved in PGC specification and cell cycle regulation, while repressing somatic gene expression [150].

Recently, a growing set of alternative experimental models has emerged to capture additional aspects of PGC specification and early development (extensively reviewed in [151, 152]). Three-dimensional pluripotent stem cell-derived models, including gastruloids and other embryo-like aggregates, recapitulate aspects of early embryonic patterning and germ layer organization, providing a spatial and temporal context in which PGC competent cells and PGC-like populations can arise alongside somatic lineages [153–160]. Recent studies utilizing these models have demonstrated hPGCLCs have recently been generated in bioengineered embryonic-like cultures that mimic peri-implantation development, where interactions with amnion-like cells and associated BMP, WNT, and NODAL signaling contribute to PGC specification [161, 162]. Organoid-based approaches and bioengineered culture systems, such as micropatterned and microfluidic platforms, further enable controlled manipulation of signaling gradients and cell–cell interactions to understand how physical cues influence PGC induction [163–165]. The addition of basement membrane extract with BMP4 enhances hPGCLC differentiation, highlighting an important role for the extracellular matrix in PGC specification [166]. A subsequent study demonstrated that co-culture with reconstituted fetal ovaries or three-dimensional amnion-like cell cultures promotes hPGCLC maturation, highlighting the importance of cellular niches in germ cell development [167]. In addition, ex vivo culture of embryonic tissues and gonadal explants has served as a complementary strategy for studying PGCs within a native tissue environment [168–172].Together, these studies established a spectrum of in vitro models that have been instrumental in dissecting the molecular determinants of PGC specification and germ cell development.

While these advances have established a strong foundation for studying PGC specification in vitro, important questions remain regarding how faithfully PGCLCs recapitulate the epigenetic state of endogenous PGCs. One of the defining features of germ cell development is extensive epigenetic reprogramming, including genome-wide DNA demethylation, imprint erasure, and repression of transposable elements. While PGCLCs reproduce many aspects of this process, several studies have reported incomplete or heterogeneous epigenetic resetting in vitro. For example, genome-wide analyses of PGCLCs have revealed incomplete erasure of parental imprints and persistent DNA methylation at specific genomic loci compared to in vivo PGCs [173,174].In addition, certain transposable element families and germline regulatory regions exhibit aberrant methylation patterns in PGCLCs, suggesting that the timing and coordination of epigenetic reprogramming may not be fully recapitulated under in vitro conditions [174]. These findings highlight that while PGCLCs capture many transcriptional features of PGC identity, their epigenetic fidelity remains variable. The potential epigenetic infidelity of PGCLCs has important implications for both basic research and potential translational applications of in vitro gametogenesis. Because germ cells transmit genetic and epigenetic information to the next generation, incomplete epigenetic resetting can lead to abnormal imprinting, transposon activation, or heritable epimutations. Consequently, continued investigation into the epigenetic integrity of PGCLCs is essential (Table 1).

## Conclusion and perspective

PGC specification is best understood not as a linear pathway driven by a few master regulators, but as an integrated process in which signaling pathways, transcriptional networks, epigenetic remodeling, transposable element repression, and metabolic programming act in concert to establish germ cell identity. While canonical factors such as PRDM1, PRDM14, TFAP2C, and SOX17 initiate PGC specification, recent work highlights a broader regulatory network in which competence factors, chromatin regulators, and signaling modulators dynamically interact to stabilize germ cell identity while suppressing somatic programs. A defining feature of this transition is extensive epigenetic reprogramming, including genome-wide DNA demethylation, histone remodeling, imprint erasure, and tightly controlled repression of transposable elements. Importantly, these processes are not merely downstream consequences of PGC specification but are actively regulated by PGC determinants and cellular metabolism. Metabolic intermediates such as *α*-KG, SAM, and acetyl-CoA directly influence the activity of epigenetic enzymes, linking metabolic state to chromatin remodeling.

These integrated mechanisms help explain a long-standing observation in the field: while in vitro PGCLC systems have achieved remarkable success, their efficiency and fidelity remain variable. Most in vitro approaches rely heavily on cytokine-based induction, particularly BMP4 supplementation, yet many of these models cannot fully recapitulate the spatial gradients or timing of competence windows present in vivo. Transcription factor-driven systems can bypass certain cytokine requirements, but incomplete epigenetic reprogramming or aberrant metabolic states may limit long-term developmental competence. Thus, improving in vitro gametogenesis will likely require coordinated manipulation of signaling pathways, transcriptional networks, chromatin modifiers, and metabolic conditions.

The translational implications of these findings are profound. In vitro-generated gametes hold significant promise for treating infertility, modeling germline diseases, preserving fertility in cancer patients, and potentially restoring reproductive capacity in individuals lacking functional germ cells. However, germ cells are unique among cell types in that their genetic and epigenetic states are transmitted to the next generation. Incomplete imprint erasure, aberrant DNA methylation patterns, or insufficient repression of transposable elements could introduce heritable epimutations with long-term developmental consequences. Even subtle perturbations in chromatin state or metabolic programming during in vitro specification may influence offspring health. Therefore, the benchmark for success of in vitro gametogenesis cannot be limited to morphological or transcriptional resemblance; it must include rigorous assessment of epigenetic fidelity, imprint integrity, and transgenerational stability. Such insight will be essential not only for refining our understanding of mammalian reproductive biology but also for ensuring that in vitro-derived gametes are produced with the safety, fidelity, and developmental competence required for clinical translation.

## Figures and Tables

**Figure 1.**
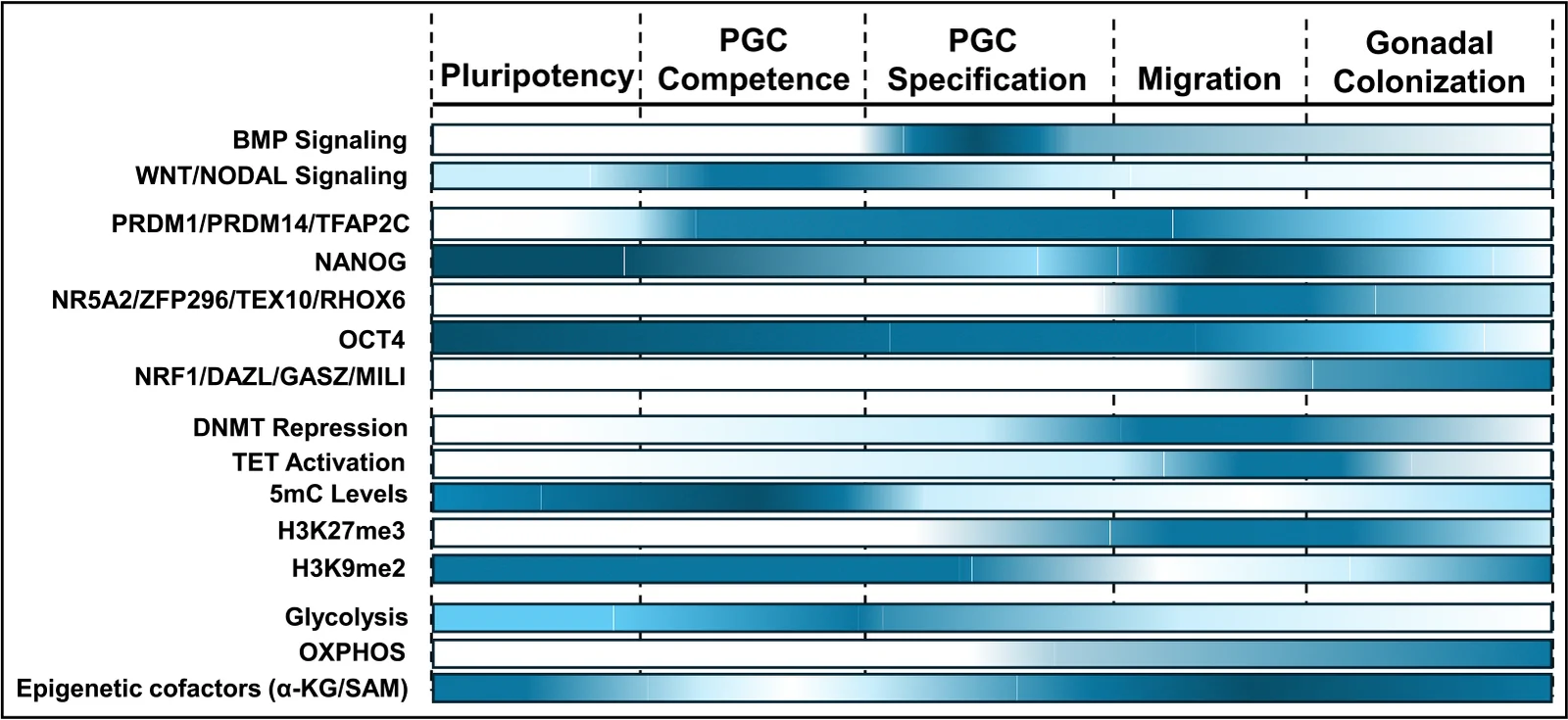
Comparison of mouse and human PGC transcription regulatory networks. In mice, BMP4 and WNT3 signaling activate SMAD1/5/8 and *β*-catenin/T (Brachyury), which converge on the core germline regulators PRDM1, PRDM14, and TFAP2C while suppressing somatic differentiation. In humans, WNT/NODAL and BMP4 signaling promote germline specification through EOMES, SOX17, GATA3, TFAP2C, and PRDM1. Solid arrows indicate established regulatory interactions, whereas dashed arrows represent proposed mechanisms. Human regulatory interactions are predominantly based on in vitro PSC differentiation models.

**Figure 2.**
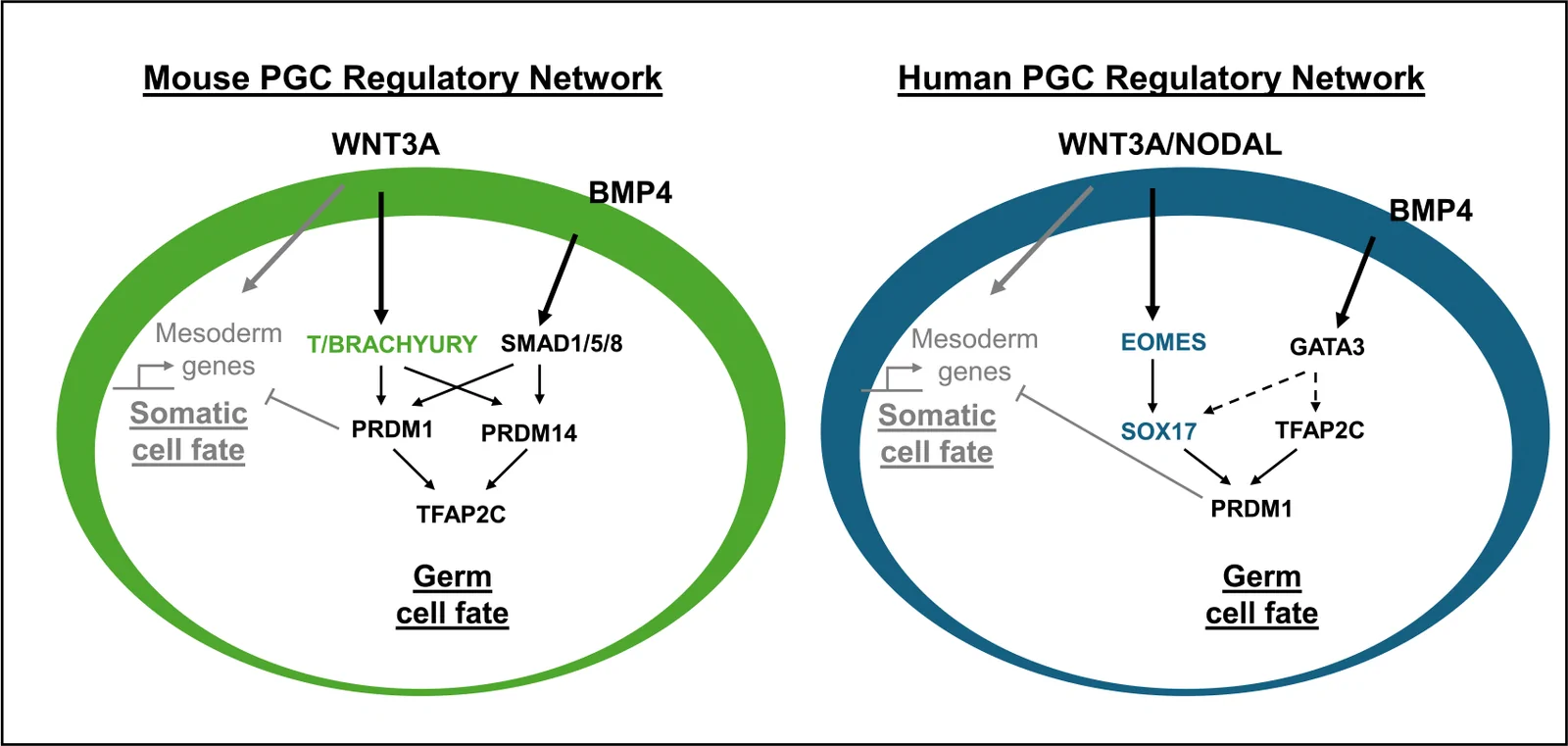
Integrated regulatory dynamics during primordial germ cell development. Schematic overview of the coordinated signaling, transcriptional, epigenetic, and metabolic programs that govern the transition from pluripotency through germline competence, specification, migration, and sex-specific differentiation. WNT/NODAL signaling establishes germline competence, while BMP peaks during specification to initiate the core germline transcriptional program (PRDM1/PRDM14/TFAP2C), accompanied by dynamic regulation of NANOG and the somatic gatekeeper OTX2. Network modulators (NR5A2/ZFP296/TEX10/RHOX6) stabilize germline identity during migration, followed by OCT4/DAZL/NRF1/GASZ to regulate PGC survival and proliferation during gonadal development. Specification and migration coincide with DNMT repression, TET activation, progressive loss of global DNA methylation (5mC), and dynamic changes of histone modifications. Metabolic preference shifts from glycolysis toward oxidative phosphorylation, which alters availability of epigenetic cofactors (e.g. *α*-ketoglutarate and S-adenosylmethionine). Gradient intensity reflects relative activity or abundance at each stage.

**Table 1.** Comparison of primordial germ cell development in vivo and pluripotent stem cell–derived PGC-like cells in vitro

Feature	In vivo PGC development	In vitro PGCLC models	Key strengths and opportunities	Representative references

Inductive signals	Spatially restricted BMP, WNT, and NODAL gradients establish PGC competence and specification	Defined cytokine combinations (BMP4, WNT agonists, Activin/NODAL) robustly induce PGCLCs	Successfully recapitulates core inductive signaling logic; future refinement may incorporate spatial patterning	[5, 175, 176]
Developmental competence	Spatially restricted epiblast cells acquire PGC competence during a defined developmental window	Stepwise differentiation through EpiLC or mesoderm-like intermediates mimics developmental progression	Faithfully models competence transition; variability across PSC lines suggests opportunity for optimization	[5, 8, 177]
Core transcription factors	Sequential activation of PRDM1/PRDM14/TFAP2 C (mouse) or SOX17-centered network (human)	Cytokine-driven or TF-induced systems activate canonical germline regulators	Demonstrates sufficiency of core transcriptional network; offers powerful platform to dissect regulatory hierarchy	[7, 14, 178]
Expanded transcriptional network	Stage-specific modulators (NR5A2, ZFP296, TEX10, etc.) stabilize germline identity	Emerging incorporation of additional regulators into differentiation systems	Provides opportunity to enhance stability and fidelity of PGCLCs	[26, 42]
Global epigenetic reprogramming	Genome-wide DNA demethylation during migration resets the germline epigenome	PGCLCs undergo substantial DNA demethylation	Captures major reprogramming dynamics; locus-specific differences remain an area for refinement	[60, 179]
Imprint regulation	Precisely timed imprint erasure and sex-specific methylation	Partial or asynchronous imprint erasure observed in PGCLC systems	Enables mechanistic study of imprint control; improved timing may enhance fidelity	[76, 179, 180]
Chromatin regulation and genome stability	Coordinated histone remodeling and transposable element repression maintain genomic integrity during hypomethylation	Key chromatin and TE repression pathways are active in PGCLCs	Provides a system to study chromatin–genome stability interactions; long-term stability should be studied	[78, 181, 182]
Metabolic remodeling	Transition from glycolysis-dominant epiblast to OXPHOS-enriched migratory PGCs	Metabolic shifts observed during in vitro differentiation	Offers opportunity to experimentally manipulate metabolic–epigenetic regulation	[132, 135]
Microenvironment	Interaction with somatic embryonic and gonadal niche	2D and 3D aggregate systems partially recreate cell–cell interactions	Incorporation of microenvironment cues may further enhance formation and fidelity	[157, 183]
Functional competence	Full gametogenesis and transmission to offspring (mouse)	Mouse PGCLCs can generate functional gametes	Demonstrates remarkable developmental potential; long-term safety and epigenetic stability remain active areas of research	[6, 184]

This table summarizes key regulatory features of PGC specification across signaling, transcriptional,epigenetic, metabolic, and functional mechanisms. In vitro PGCLC systems successfully recapitulate the core inductive signaling pathways and transcriptional networks that define germline fate, as well as major aspects of epigenetic reprogramming and developmental progression. At the same time, differences in spatial context, imprint dynamics, metabolic remodeling, and niche interactions highlight opportunities to further refine model fidelity. Representative references supporting each comparison are indicated.

## Data Availability

Not applicable.
